# Convicting Peaceful Protesters: Proportionality’s Proper Place at Criminal Trial

**DOI:** 10.1093/ojls/gqae009

**Published:** 2024-04-03

**Authors:** Richard Martin

**Keywords:** human rights, proportionality, peaceful protest, European Court of Human Rights, criminal trial

## Abstract

Suppose that a defendant’s conviction would amount to an interference with their right to peaceful protest, protected by articles 10 and 11 of the European Convention on Human Rights. Is a court then obliged to make a conviction turn on a fact-sensitive proportionality assessment justifying the interference? Drawing on the jurisprudence of the domestic and Strasbourg courts, this article argues that the case law has crystallised into two paradigms that provide distinct answers: the ‘justificatory paradigm’ in European human rights law and the ‘offence-centric’ paradigm in domestic law. The article exposes how and why this divergence has developed, what is at stake at the level of constitutional values and how this conflict might be resolved. It is argued that compliance with Strasbourg now depends on the integration of the justificatory paradigm into domestic law. The article imagines how this might be done in a manner sensitive to domestic constitutional values, using the mechanics on offer in the Human Rights Act 1998.

## 1. Introduction

The task of this article is to disentangle a knotty legal question reaching the highest courts, but yet to be confronted in the scholarship: must a criminal court conduct a fact-sensitive proportionality assessment where a defendant’s conviction for a public order offence would amount to an interference with the right to peaceful protest?^1^ The article’s central claim is that this question is being answered differently by the European Court of Human Rights (ECtHR, Strasbourg) and the domestic courts, including the UK Supreme Court. The ECtHR requires courts to assess a conviction’s proportionality in a broader class of cases than the highest domestic courts are willing to recognise. The knot has only tightened with the Supreme Court’s insistence that domestic law *is* consistent with Strasbourg.^2^ The consequence, it is argued, are convictions for public order offences which are in violation of the procedural duty Strasbourg has implied into article 10 (the right to freedom of expression) and article 11 (the right to freedom of peaceful assembly) of the European Convention on Human Rights (ECHR, the Convention).

The question is a knotty one, in part, because to answer it, one must disentangle the bodies of criminal law, human rights law and public law that have been interwoven in different ways by the ECtHR and domestic courts. But it is also complex, because how these bodies of law are prioritised depends on courts’—often implicit—reading of foundational principles concerning the courts’ very function in human rights-based adjudication, the deference to be shown to the legislature and the very nature of the criminal conviction. The article is an endeavour to loosen the legal knots the question of convicting peaceful protestors has given rise to—that is, to expose how and why a sizeable body of conflicting jurisprudence has emerged, what is at stake as a matter of constitutional principle and how it might be resolved given the complexities the question involves.

To these ends, the article advances three arguments. First, a conflict between the ECtHR and domestic courts *does* exist, despite claims by the Supreme Court to the contrary. In doctrinal form and constitutional implication, two distinct legal paradigms now operate: one authored by the ECtHR (the ‘justificatory’ paradigm’), the subject of section 3; the other crafted by the domestic courts (the ‘offence-centric’ paradigm), the focus of section 4. Second, the basis for this divergence is deceptively simple. It appears based on domestic courts’ preference for one line of Strasbourg authority (on ‘general measures’) over another (on the procedural duties of articles 10 and 11). Scratch the surface, though, and deeper constitutional fault lines emerge. These concern the significance of proportionality as a device for courts to assess justifications for specific exercises of state power; the legitimate role of the legislature in conclusively striking the balance between civil rights and public order; and the criminal court’s status as a public authority when it decides whether to convict a defendant. Third, despite this divergence and its complexities, there is a means of integrating the justificatory paradigm into domestic law so as to achieve compliance with Strasbourg *and* remain sensitive to the principled concerns identified. Section 5 sketches out what this reconciliatory approach would look like, inspired by adaptions to the proportionality test made by the Supreme Court once before in the analogous situation of tenants’ evictions by public authorities.

## 2. The Strands of the Question

It is helpful to begin by setting out the three strands of law that explain whether, and if so how, the rights of the accused under articles 10(1) and 11(1) come to be considered at trial in the first place. The first concerns how the same protest-related activity is classified in different ways: human rights law offering *prima facie* protection of it, criminal law prohibiting and punishing it. The ECtHR has included a raft of non-violent protest activities within articles 10(1) and 11(1) (discussed below). At the same time, in England and Wales an increasing number of these same activities are being criminalised by public order offences that seek to target disruptive protests.^3^ The offences range from those of general application (eg aggravated trespass^4^ and criminal damage^5^) to offences that specifically target protest techniques. The latter include manner and form conditions imposed on assemblies and processions by police (breach of which is an offence), including conditions on ‘slow-walking’^6^ and noisy forms of protest,^7^ but also new standalone offences such as ‘locking-on’ to objects or fellow protestors, obstructing major transport works and interfering with national infrastructure.^8^

The second strand relates, in turn, to what this overlap means for the interaction between criminal law and human rights law within the criminal process. Specifically, it concerns how the classification of the defendant’s behaviour affects what a court must be satisfied of, or able to justify, when determining whether to convict a peaceful protestor of a particular public order offence. As a matter of criminal law, to convict, the court must be sure of the defendant’s guilt, based on the prosecution having proven the elements of the offence to the criminal standard of proof, and that no defences provided for in the offence are satisfied. As a matter of human rights law, however, a conviction for protest activity falling within articles 10(1) and 11(1) is a distinct form of interference imposed by the court. It must, therefore, be justified as ‘necessary in a democratic society’ on the basis provided for in article 10(2) or 11(2).

The ‘necessary in a democratic society’ justification involves four steps that collectively form the doctrine of proportionality: (i) the interference must be in pursuit of a legitimate goal; (ii) there must be a suitable or rational connection between the policy and the goal; (iii) there must not be a less restrictive but equally effective alternative means to further the legitimate aim; and (iv) fair balance must be struck between the seriousness of the interference of the right against the importance of the competing right or interest.^9^ In practice, much turns on stage (iv), referred to as proportionality *sensu stricto.*^10^ It requires the court to consider a series of highly context-specific, evaluative questions: what was the duration of the protest? To what extent were the rights of others actually interfered with? Was any disruption targeted as the object of the protest? Did the protest relate to views which many would see as being of considerable breadth, depth and relevance? Was the protest entirely peaceful? Did anyone submit a complaint about the protest activity? Did the protestor’s behaviour involve any further offences?^11^

Without any express requirement in the offence for its application to be proportionate to the defendant’s protest rights protected by articles 10 and 11, the potential arises for the court to convict the defendant on the basis of the elements of the offence having been proven—but without having justified, or even considered, the conviction’s proportionality based on the evaluative questions just listed. Faced with this, where the offence contains a ‘reasonable’ or ‘lawful’ excuse defence, the courts have used this defence as a textual basis to read in a requirement that the conviction be proportionate.^12^ But what does a criminal court do where the offence does not provide for a ‘reasonable’ or ‘lawful’ excuse? Where does the conduit for the proportionality assessment come from? Is it really necessary for every conviction, as a form of rights interference, be justified on the facts of a specific case? Ultimately, what is the priority between criminal law and human rights law?

It is these questions that have given rise to the third strand of law—the major strand out of which the two paradigms of this article emerge. One approach, adopted by Strasbourg, requires courts to justify the proportionality of a conviction on the facts of the case at hand.^13^ This, it is argued shortly, bears the hallmarks of the ‘culture of justification’: courts must demand adequate reasons for any act that affects a person’s rights.^14^ This is achieved by conducting a context-specific, evaluative proportionality assessment. The alternative approach, developed by domestic courts and constituting the current law, makes it possible for a conviction interfering with the defendant’s article 10 and 11 rights to be justified without a proportionality assessment. This arises where the courts deem the legislature to have struck the balance between competing rights when it devised the offence at the legislative stages.^15^

On this latter account, a conviction is cast as a manifestation of a policy choice by Parliament to criminalise behaviour which has been debated, assessed and justified according to values and procedures within the political constitution. This negates the need for judicial assessment of the proportionality of the offence’s application to the defendant’s case unless the legislature incorporated a proportionality assessment into the offence or the court considers the proof of the ingredients of the offence are insufficient to ensure the conviction’s proportionality.^16^ This is a kind of domestic version of the ‘general measures’ approach adopted by the ECtHR in *Animal Defenders*, where the ECtHR will take account of the adequacy of domestic administrative, legislative and adjudicative processes to permit rights-interfering laws applied to predefined situations, regardless of individual facts.^17^

With the aspects of law that interact in order to answer the fundamental question addressed in this article now sketched out, the following sections proceed to explore the two paradigms that have emerged in answer to it.

## 3. The Justificatory Paradigm at Strasbourg

In a ‘culture of justification’, the judiciary’s role is to demand that every act of the state that affects a person is justified to him or her with strong enough reasons.^18^ The culture of justification has been grounded in a conception of liberal-democratic constitutionalism,^19^ and of persons as justificatory agents whose status allows them to challenge and demand adequate reasons for any law or act that relevantly affects them.^20^ In institutional terms, courts must go beyond their conventional role of applying rules and interpreting principles to assessing justifications.^21^ The proportionality test has become *the* device by which the justifiability of state action is assessed in countries with constitutional structures that give rise to a culture of justification.^22^ In this section, it is argued that the ECtHR’s jurisprudence of articles 10 and 11 has the hallmarks of the culture of justification because of: (i) the scope of the rights that trigger the justificatory requirement; (ii) the requirement of domestic courts to assess justifications through proportionality; and (iii) the association of peaceful protest with liberal-democratic constitutionalism that grounds the culture of justification.

### A. The Scope of the Right to Peaceful Protest

The ‘right to peaceful protest’ is an amalgamation of the protections offered by articles 10 and 11.^23^ Article 10 is considered by the ECtHR to be a *lex generalis* in relation to article 11 because article 10 protects not only the substance of ideas and information, but also the means by which they are conveyed.^24^ In recent years though, the ECtHR has grown more receptive to assessing interferences with peaceful protest under article 11 and finding violations of it.^25^ Both articles adopt the two-stage structure of qualified rights: the first, identifying whether the right is engaged/interfered with; the second, assessing the state’s justification for this. As observed by Cohen-Eliya and Porat, a defining feature of the culture of justification is courts’ tendency to read constitutional provisions, including bills of rights, in a way that allows, if not requires, courts to focus on the justifiability of the act in question. This is achieved by relying on a breadth of substantive values and goals to broaden the scope of the right at stage one, enabling the court to assess justifications at stage two.^26^ This is evident in the article 10 and 11 jurisprudence of the ECtHR in two respects.

First, the ECtHR has proven very open to affording disruptive but peaceful modes of protest *prima facie* protection under articles 10(1) and 11(1). Aside from where protestors have violent intentions, incite violence or otherwise reject the foundations of a democratic society,^27^ the disruptive protest activity falling within the scope of articles 10(1) and 11(1) now includes: breaking into a construction site, climbing into trees, obstructing machinery to impede engineering works by environmental activists;^28^ using ‘go-slow’ walking techniques to repeatedly and intentionally block a public highway;^29^ protesting in a courthouse by chanting slogans, displaying banners and throwing leaflets for an hour, impeding hearings;^30^ blocking major roads for approximately two days, creating significant road delays and cross-border trade disruption in protest over agricultural subsidy reforms;^31^ and impeding the scheduled eviction of squatters, the foreseeable result of which was to impede the activities of others.^32^

Second, the ECtHR has taken control over what constitutes ‘peaceful assembly’ in article 11(1) by classifying the term as an autonomous one.^33^ Consequently, what constitutes protest activity for the purposes of article 11(1) is not dependent on how it is categorised in domestic law. This ‘serves the interests of the protection of the right against improper classifications in national law’^34^ and enables the ECtHR to require justification for a greater range of non-violent protest activity. As expressed by Strasbourg, the ‘unlawfulness’ of an event does ‘not give carte blanche to the authorities; the domestic authorities’ reaction to a public event remains restricted by the proportionality and necessity requirements of Article 11 of the Convention’.^35^ A recent example of the autonomous principle in action is *Laurijsen and Others v the Netherlands*. In finding a breach of article 11(1) because the Dutch Supreme Court did not give relevant and sufficient reasons for the conviction on the facts of the case, the ECtHR was particularly critical of the domestic court’s failure to examine whether the assembly was ‘peaceful’ according to the ECtHR’s autonomous meaning, irrespective of Dutch law’s classification of the protest as unlawful.^36^

### B. The Emergence of the Justificatory Duties Placed on Courts

Under the culture of justification, great trust is placed in the judiciary as an institution capable of imposing ‘rationality and reasonableness on other authorities’.^37^ This is achieved by demanding public authorities give reasons for every act plausibly regarded as violating a right, and then assessing whether the reasons given can be adequality justified using the proportionality test. This section traces two lines of Strasbourg authority which, it is argued, demonstrate that the ECtHR has read into articles 10 and 11 the kind of justificatory demand and assessment integral to the culture of justification. The analysis reveals the gradual incorporation of the ECtHR’s own standards of scrutiny of national authorities into those expected of national courts when assessing convictions that engage articles 10 and 11. The result is that a domestic court must ensure the proportionality of a conviction based on the circumstances of the case. Very little has been written about the existence, let alone development, of this procedural component of articles 10 and 11. This warrants exploring the jurisprudence in some detail.

The first line of authority concerns peaceful protest cases assessed under article 10. In the early case of *Handyside v UK*, Strasbourg established that its supervision of whether an interference is ‘necessary in a democratic society’ must examine national courts’ decisions ‘in the light of the case as a whole’ and that the ECtHR must determine for itself ‘whether the reasons given by the national authorities to justify the actual measures … are relevant and sufficient under Article 10, para 2’.^38^ In *Sunday Times v UK*, Strasbourg stated that it was insufficient that an interference be imposed because its subject matter ‘fell within a particular category or was caught by a legal rule formulated in general or absolute term’.^39^ The ECtHR had to be satisfied the interference was necessary ‘having regard to the facts and circumstances prevailing in the specific case before it’.^40^ For years, this formulation was repeatedly stated and applied. Crucially, though, in the case of *Oberschlick v Austria*, which concerned the limits of acceptable criticism in the context of public debate on a political question of general interest, Strasbourg extended its own approach to scrutiny to that expected of national authorities when conducting their own review. Strasbourg stated:

In such cases the Court has to satisfy itself that the national authorities did *apply standards which were in conformity with these principles* and, moreover, that in doing so they based themselves on an *acceptable assessment of the relevant facts*.^41^

These twin procedural requirements of applying the principles of articles 10 and 11 and doing so based on the relevant facts of the case went on to be applied in a series of article 10 cases throughout the 1990s.^42^

It was not until much later, in *Perinçek v Switzerland*, that the scope of these procedural duties was elaborated on by the Grand Chamber of the ECtHR.^43^ In *Perinçek*, the Vaud Cantonal Court convicted the applicant, the chairman of the Turkish Workers’ Party, for publicly denying the genocide against the Armenian people. The Grand Chamber was highly critical of the Vaud Cantonal Court’s silence on the effect of the conviction on the applicant’s rights under article 10, in particular that it ‘made no reference to the conviction’s necessity in a democratic society, and did not engage in any discussion of the various factors that bear on that point’.^44^ When proposing the offence with which the applicant was convicted, the Swiss government had alluded to the potential conflict between freedom of expression and criminal convictions. But in what can be read as a broader point about the nature of the procedural duty on domestic courts, the Grand Chamber stated:

an interference with the right to freedom of expression that takes the form of a criminal conviction *inevitably requires detailed judicial assessment* of the specific conduct sought to be punished. *In this type of case*, it is normally not sufficient that the interference was imposed because its subject matter fell within a particular category or was caught by a legal rule formulated in general terms; what is rather required is that it was necessary in the specific circumstances.^45^

This was the first time Strasbourg required not just national authorities to apply standards in conformity with Convention principles, but for national courts specficially to engage in a detailed *judicial* assessment of the *specific conduct*. The requirement for domestic courts to undertake scrutiny of the interference under article 10 in the context of a criminal conviction was applied in *Saygılı and Seyman v Turkey*. In that case, Strasbourg stated that the ‘quality of the judicial review in respect of the necessity of the measure is of particular importance in the context of proportionality assessment under Article 10’.^46^

The procedural duty on domestic courts has been further developed in two very recent protest cases involving article 10. In *Handzhiyski v Bulgaria*, the applicant’s conduct was an act of ‘minor hooliganism’ in which he publicly mocked a monument of an early 20th-century political figure on Christmas Day by placing a Santa Claus cap and a red bag on it.^47^ The monument was of the founder of a political party which was providing the main parliamentary support for the Bulgarian government of the day. The applicant’s act formed part of a prolonged nationwide protest against the government. The ECtHR in *Handzhiyski* affirmed the Grand Chamber’s reasoning in *Perinçek*. The ECtHR emphasised that the applicant’s conduct was a symbolic mode of expression, imparting ideas about the government, and a mode of engagement in political protest. Where an interference takes the form of a penalty, the Court continued ‘it *inevitably* calls for a detailed assessment of the specific conduct sought to be punished. It cannot normally be justified solely because the expression at issue was caught by a legal rule formulated in general terms.’^48^ The assessment of proportionality was especially ‘nuanced’, the ECtHR explained, in circumstances like those in *Handzhiyski*, where there was no damage to the monument: ‘In such situations, the precise nature of the act, the intention behind it, and the message sought to be conveyed by it cannot be matters of indifference.’^49^

A similar approach was followed in the case of *Bumbeș v Romania*.^50^ The applicant handcuffed himself to barriers blocking access to the parking area of a government building to protest against a controversial mining project. The Romanian courts found him guilty of a public order offence and the failure to provide the requisite notice to the authorities. Although the interference had been lawful and in pursuit of a legitimate aim, Strasbourg found a violation of article 10 on the basis that the domestic courts had failed to satisfy their procedural duty to assess the proportionality of the conviction. It is worth citing in full Strasbourg’s emphasis in its judgment on the need for national courts to look beyond the criteria of the domestic law to probe whether, on the facts of the specific case, the application of the offence struck a fair balance:

when dismissing the applicant’s challenge against the police report and the fine imposed on him, the national courts did not assess the level of disturbance his actions had caused, if any. They merely observed that the applicant had failed to comply with the prior-declaration requirement … the proportionality principle demands that a balance be struck between the requirements of the purposes listed in Article 11 § 2 on the one hand, and those of free expression by word, gesture, or even silence by persons assembled on the streets or in other public places on the other [ref omitted]. Nevertheless, the Court notes that the national courts did not seek to strike this balance giving the preponderant weight to the formal unlawfulness of the event in question.^51^

It was not enough, the ECtHR insisted, for domestic courts to simply apply the elements of the public order offence itself—they must seek to strike the balance based on the particular disturbance caused to others based on the specific facts of the case at hand.

The second line of authority is similar to that just traced, only it has emerged in protest cases under article 11. In *United Communist Party of Turkey and Others v Turkey*, Strasbourg read the twin procedural duties from article 10 across to article 11, which requires national authorities to (i) apply standards which are in conformity with the principles of article 11 and (ii) do so on the basis of an assessment of the various facts in the specific case that bear on the conviction’s necessity in a democratic society.^52^ In protest cases since the mid-2010 onwards, Strasbourg has emphasised that any large-scale gathering in a public place will inevitably create inconvenience for the population or some disruption to ordinary life, including the disruption of traffic. As such, when determining whether an interference is proportionate or not, the degree of tolerance expected of national authorities ‘cannot be defined in the abstract: one must look at the particular circumstances of the case and particularly at the extent of the “disruption to ordinary life”’.^53^

There has been a notable shift in focus in this jurisprudence from what national authorities generally must do to satisfy article 11 to what domestic courts specifically must do in order to comply with article 11. Given that courts are institutions uniquely well placed to examine interferences with rights on a case-by-case basis, certainly in a way that cannot be done by legislatures when devising offences of general application, the direct application by the ECtHR of the procedural duties under article 11 to national courts specifically make sense. This shift has come in four very recent cases in which the ECtHR has found violations of article 11, including cases involving domestic ‘manner and form’ restrictions placed on protest activities.^54^ In these cases, the basis of the breach of article 11 has been domestic courts’ failure to comply with the procedural duties. The breach has arisen from either the proportionality assessment not being performed because the domestic court gave preponderant weight to the formal unlawfulness of the conduct in domestic law or because the domestic court’s assessment omitted important aspects of the applicant’s specific protest-related behaviour in its evaluation of proportionality *sensu stricto*.

Of these recent cases, one which crystallises the implications of article 11’s procedural limb in the protest context particularly clearly is *Öğrü and Others v Turkey*.^55^ The applicants participated in several demonstrations in the city of Adana, including commemorating the massacre of leftist guerrillas in 1972 and protesting against tuition fees for higher education. During the demonstrations, hundreds of people gathered, marched, held placards, chanted slogans and occasionally blocked road traffic. The applicants were convicted and fined for public order offences relating to the demonstrations. Strasbourg observed how the scope of the supervisory control carried out by the domestic court was limited to verifying the accuracy of the charges against the applicants and, crucially, had failed to balance the applicants’ right to peaceful demonstration on the one hand with the maintenance of public order and the protection of the rights of others on the other. In the absence of such a balancing exercise, the domestic courts had failed to provide relevant and sufficient reasons to establish that the interference with applicants’ right was ‘necessary in a democratic society’. This failure alone was sufficient to violate article 11. The ECtHR drew a specific analogy with the procedural requirement of national courts to perform a proportionality assessment in its article 8 jurisprudence:

it [the Court] has already found a violation of Article 8 of the Convention because the courts had failed, inter alia, to rule on the proportionality of the interference (*Zehentner v. Austria* and *Bjedov v. Croatia*). For the Court, a similar reasoning must apply to Article 11 of the Convention.^56^

What was the reasoning in these two article 8 cases being read across to article 11? In *Zehentner*, the ECtHR held there had been a violation of article 8 because, *inter alia*, there had not been the possibility to have the proportionality of the measure—the dispossession of the applicant’s home under domestic housing law—determined by the courts.^57^ Similarly, in *Bjedov*, the violation of article 8 was linked to the inability of the applicant to challenge the proportionality of her eviction before an independent tribunal.^58^ In summary, the ‘similar reasoning’ Strasbourg was referring to in *Öğrü* must, it is argued, be that where the right to peaceful assembly is engaged, domestic courts must have assessed the proportionality of a conviction, involving a fact-specific evaluation.

### C. Grounding the Requirement for Justification

Why should courts be empowered to demand and assess justifications? Further still, why should they do so using the proportionality criteria, which involve empirical and moral questions on trade-offs compatible with democracy? A lively body of public law scholarship has sought to provide a normative and instrumental foundation for the culture of justification that address these core questions.^59^ One of the most influential accounts is Mattias Kumm’s. Because it integrates the culture of justification within an analysis of liberal democracy and political participation, it has particular resonance in the context of peaceful protest. In order for law to be constitutionally legitimate, Kumm argues, the political process must reflect political equality and be based on majoritarian decision making. But that alone is not enough, Kumm insists. It must be accompanied by outcome-orientated criteria aimed particularly at those left worst of and heavily affected by legislation. Those burdened by legislation must ‘be able to interpret the legislative act as a reasonable attempt to specify what citizens—all citizens, including those on the losing side—owe to each other as free and equals’. The outcome must ‘plausibly qualify as a collective judgment of reason about what the commitment to rights of citizens translates into under the concrete circumstances addressed by the legislation’.^60^ Proportionality is the legal device, Kumm argues, for facilitating this process of interpretation and for assessing whether outcomes do plausibly qualify in such terms. This is why Kumms claims proportionality-based review of interference with rights is of equal importance as the right to vote.^61^

Applied to non-violent protest, the justification requires the translation of protest rights to concrete situations, often involving minority groups motivated by genuine concerns of public interest—the climate crisis, social equality, international rights abuses—whose actions are criminalised by public order offences. The outcome, if it takes the form of a conviction, must be justified in terms that an offender might reasonably accept, even if they disagree with the result.^62^ The association with peaceful protest and liberal democracy is one routinely made by the ECtHR.^63^ A specific aim of article 11, according to the ECtHR, is to secure a forum for public debate and the open expression of protest.^64^ The ability to express opinions on matters of public interest beyond party politics or elections, Strasbourg has observed, invigorates participation in public life, promotes a culture of open democracy and offers a means of holding corporations, public authorities and the government to account.^65^ In making sense of the culture of justification in the context of Europe, Cohen-Eliya and Porat draw particular reference to the ‘suspicion towards popular democracy’ after the disintegration of the young democracies of the early 20th century.^66^ This explains, in part, a preference for recognition and protection by the courts of rights ‘which do not derive their legitimacy from popular support, but from professionalism, rationality and coherency’.^67^

To this democratic foundation for the culture of justification we can add a more straightforwardly instrumental one offered by Kumm which again applies well to public order law and protest rights. Judicially assessed justifications can, he argues, improve outcomes by addressing distinct types of pathologies that can vitiate the democratic process, even in mature liberal democracies. The pathology most salient here is what Kumm describes as ‘hyperbole and ideology’: the tendency for what are, in principle, legitimate concerns and reasons to be invoked by the government, but without being ‘appropriately tailored to engage the realities on the ground’—that is to say, they are not a result of a judicious discernment of the facts or the weighing of competing concerns in a contextually sensitive way.^68^ The Joint Committee on Human Rights has warned of precisely such a pathology. It is ‘naïve’, the Committee has stated, to assume ‘every potential clash of interests raised before the courts, possibly many years down the line, was anticipated and considered during a Bill’s passage through Parliament’.^69^ Similarly, the Law Society observed that

even with the best of intentions, an Act of Parliament may have unintended, unduly harsh consequences for a particular person or class of people. Balancing rights issues against other factors—as our courts are experts in doing—is highly dependent on the facts and context of the case.^70^

There is good reason for scepticism about whether Parliament sought to balance competing rights, let alone consider their application in the context of peaceful protest, when crafting public order offences, especially before the Human Rights Act 1998 (HRA).^71^ The ‘hyperbole and ideology’ pathology, Kumm observes, tends to arise in the context of security concerns relating to crime and disorder where ‘the pay-off for the public authorities in power and the security apparatus in particular in terms of gaining discretionary power is great and the risks of abuse or mistake are seemingly restricted to relatively circumscribed minority groups’.^72^ Kumm could just as well have been describing contemporary public order law making, which has seen the Home Office remarkably responsive to the calls of senior police for greater powers to target disruptive expressions of dissent. The result has been a marked broadening and deepening of public order powers, as seen in Part IV of the Police, Crime, Sentencing and Courts Act 2022 and the Public Order Act 2023.^73^ Both Acts signal the government’s pursuit of, and the majority of Parliament’s willingness to acquiesce in, a repressive criminal justice response to peaceful but disruptive protest and expressions of dissent, with a ‘significant chilling effect on civil society and the exercise of fundamental freedoms.^74^

### D. A Summary of the Justificatory Duties

This section has traced the Strasbourg jurisprudence which, it is argued, requires domestic courts, firstly, to apply standards which are in conformity with the principles of articles 10 and 11 and, secondly, to do so based on a proportionality assessment of the specific facts of the case. This is in the spirit and form of the culture of justification. The twin requirements are a constituent part of the right to peaceful protest protected by articles 10 and 11. The corollary is that Strasbourg’s assessment of whether either of these rights are violated is not confined to the substantive interference but extends to whether domestic courts have fulfilled their procedural duties. In other words, a fact-specific proportionality assessment by courts is part of the protection offered by these rights. This is distinct from, and should not be confused with, the procedural review undertaken in *Animal Defenders*, which the ECtHR used to give effect to the margin of appreciation when determining the scope of a substantive interference with the right. *Animal Defenders* is returned to below because it has been (problematically) transported into the domestic law. More immediately, the article proceeds to the domestic jurisprudence comprising the ‘offence-centric’ paradigm.

## 4. The Offence-centric Paradigm in Domestic Law

As public authorities for the purposes of section 6 of the HRA, courts must not act in a way which is incompatible with Convention rights. This is achieved through the interpretative duty in section 3(1) of the HRA, which requires courts to construe and give effect to legislation in a way which renders it compatible with Convention rights as far as is possible to do so. In a stream of judgments, domestic courts have determined the proper constitutional relationship between articles 10 and 11, sections 3 and 6 of the HRA and public order offences. This began with early freedom of expression cases arising from convictions under section 5 of the Public Order Act 1984,^75^ but has since extended to a range of public order offences,^76^ including recent convictions associated with direct action environmental and social justice protests.^77^ In this section, I draw out the essence of the case law comprising the ‘offence-centric’ paradigm, before bringing to the surface the constitutional values that underpin it.

### A. The Primacy of the Statutory Offence

The defining feature of the offence-centric parading is the use of the criminal offence as the source from which to determine whether a proportionality assessment is required to justify a conviction as a lawful interference with articles 10(1) and 11(1). Pulling together the case law, the interaction between public order offences and the right to peaceful protest are categorised in Table 1. Categories 1–2 are cases where the courts have held articles 10 and 11 are not engaged at all—these are discussed in the context of the autonomous meaning of ‘peaceful’ assembly at the end of this section. Of principle concern are the later categories. A fact-specific proportionality assessment is considered necessary in just two situations, found in category 3 and 5 offences. This leaves offences in category 4 (comprising at least four offences) and category 6 (potentially soon to compromise offences from the Public Order Act 2023—see below), where a defendant can be convicted for an offence that interferes with articles 10 and 11 without it ever being justified on the facts of the case. This section first explores the court’s creation of category 3, before examining how and why categories 4 and 6 came to be classed as distinct from it.

**Table 1.** Categories of the offence-centric paradigm

	Category 1	Category 2	Category 3	Category 4	Category 5	Category 6
Articles 10 and/or 11 ECHR engaged?	**✕**	**✕**	✔	✔	✔	✔
Do the offence’s ingredients ensure every conviction’s proportionality?	✔	✔	**✕**	✔	**✕**	✔
Is there a lawful/ reasonable excuse defence?	**✕**	✔	✔	**✕**	**✕**	✔
Proportionality assessment on individual facts of case required?	**✕**	**✕**	✔	**✕**	✔	**✕**
Examples from case law	s.68, CJPOA 1984; *DPP v Cuciurean* suggested not (at [45] & [50])	Significant criminal damage (*AG Ref. No. 1 of 2022*)	s.5, POA 1984: *Percy v DPP; Norwood v DPP; Hammond v DPP*; *Hicks v DPP* s.137, HA 1980 (*DPP v Ziegler*) Minor criminal damage (*AG Ref. No. 1 of 2022*) Covid-regulations (*Leigh v DPP*)	s.14(5), POA 1984: *James v DPP*; *DPP v Eastburn* s.5(1), Abortion Services (Safe Access Zones) Act (NI) 2023 (*SAZ*); s.9(1), POA 2023 (interference with access/provision of abortion services)	None but dicta in *SAZ* [57]	Dicta in *AG for NI* at [58]. s.1, POA 2023 (offence of ‘locking on’)? ss.6-7 POA 2023 (interfering/obstructing major transport works/national infrastructure)?

In the early judgments that comprise category 3 offences, the courts held that the proportionality of any restrictions on freedom of speech or peaceful assembly was capable of being accommodated by the express words of the statutory offence.^78^ Whether a fact-specific assessment of proportionality was required came, in effect, to depend on the whether the wording of the offence allowed for such an assessment. The key indicator used was whether the offence contained a defence of ‘reasonable’ or ‘lawful’ excuse; if so, this functioned as the conduit through which to insert a proportionality test, using section 3 of the HRA to do so.^79^ It is this reasoning that defines the category 3 cases in Table 1.

Then, in *James v DPP*,^80^ a decision followed recently in *DPP v Eastburn*,^81^ the Divisional Court held that where the offence lacks a ‘reasonable’ or ‘lawful’ excuse defence, the court need not necessarily read one into the offence. Rather, Parliament, in legislating to create the offence based on the officer’s satisfaction of the test for imposing conditions in section 14(1) of the Public Order Act 1986 (POA 1986), could—and in that particular case, should—be construed as having struck the balance itself. In the words of Ousley J in *James*, the ‘necessary balance for proportionality is struck by the terms of the offence-creating provision, without more ado’.^82^ There was no need for the court to demand, and assess on the facts of the case, the justification for the interference with the defendant’s rights.^83^ The ‘intrinsically’ proportionate class of cases, which make up category 4, was born.

The offences in category 4 include not only section 14 (and, by implication, section 12) of the POA 1986—two of the main public order powers available to police in the protest context—but also section 5(1) of the Abortion Services (Safe Access Zones) Act (Northern Ireland) 2023 (and presumably its counterpart in England and Wales, section 9 of the POA 2023). Watch this space, however. At the time of writing, the Home Office intends to amend the Criminal Justice Bill so as to remove the right to protest as a reasonable or lawful excuse to commit certain public order offences, ‘ensuring that protest is not used as a defence for criminality such as obstructing public highways, locking on, as well as public nuisance’.^84^ This would significantly expand the scope of the category 6 offences (where the offence is intrinsically proportionate, notwithstanding the presence of a reasonable excuse defence). On this article’s analysis, this would enlarge the number of convictions at risk of failing to comply with the procedural duties required of courts discussed in the preceding section.

A significant test of the offence-centric paradigm did come in a series of challenges following *Ziegler*.^85^ In *Ziegler*, the Supreme Court held that deliberate obstructive conduct with more than a *de minimis* impact remains within the scope of the right and, for the purposes of section 137 of the Highways Act 1980, the ‘lawful excuse’ element of the offence was interpreted, in accordance with sections 3 and 6 of the HRA, as requiring a proportionality assessment to ensure the conviction was not a disproportionate interference. Lady Arden JSC observed that the HRA ‘had a substantial effect on public order offences and made it important not to approach them with any preconception as to what is or is not lawful’.^86^ This was accompanied by observations that sat uncomfortably with the offence-centric paradigm. Lord Hamblen and Lord Stephens JJSC stated that prosecution and conviction, alongside arrest and sentence, were all restrictions per articles 10 and 11. Each would only be ‘necessary in a democratic society’ if justified, which requires an ‘assessment of the facts in each individual case’,^87^ with different considerations applying to each restriction.^88^ As for convictions specifically, their Lordships (with Lady Arden JSC agreeing) remarked: ‘The proportionality assessment at trial before an independent impartial tribunal depends on the relevant factors being proved beyond reasonable doubt and the court being sure that the interference with the rights under articles 10 and 11 was necessary.’^89^

These observations in *Ziegler* soon became submissions in separate appeals before the Divisional Court (*Cuciurean*), Court of Appeal (*Attorney General’s Reference No 1 of 2022*) and the Supreme Court (*SAZ*), specifically that: (i) *Ziegler* required a proportionality test to be made an ingredient of any offence which impinges on the exercise of rights under articles 10 and 11; (ii) since a conviction for any offence arising out of a peaceful protest involves a restriction on articles 10 and 11, the prosecution must prove that the conviction would be justified and proportionate; and (iii) *Ziegler* supported the argument that the duty of a criminal court to consider the proportionality of a conviction where Convention rights are engaged arises from section 6(1) of the HRA.

These submissions failed and the offence-centric paradigm was reaffirmed. A clear analysis to this effect was provided by Lord Burnett CJ in *Cucuirean*. After stating that the reasoning in *Ziegler* was expressed solely in the context of the lawful excuse defence in section 137 of the Highways Act, his Lordship continued:

[I]t is impossible to read the judgments in *Ziegler* as deciding that there is a general principle in our criminal law that where a person is being tried for an offence which does engage articles 10 and 11, the prosecution, in addition to satisfying the ingredients of the offence, must also prove that a conviction would be a proportionate interference with those rights.^90^

As for the attempt by the appellants to ground a requirement to consider the proportionality of a conviction as part of the court’s duties under section 6 of the HRA, Lord Burnett CJ was clear that section 6 only applied if proportionality was an ingredient of the offence in the first place—‘it depends on the substantive law governing the offence’.^91^*Cuciurean* was supported by the Court of Appeal in *AG’s Ref No 1 of 2022* and the Supreme Court in *SAZ*.

The latter of these appeals, *SAZ*, concerned clause 5(2)(a) of the Abortion Services (Safe Access Zones) (Northern Ireland) Bill, now enacted, which criminalises anti-abortion protests and other specified behaviour within ‘safe access zones’ around abortion clinics; the offence does not include a reasonable excuse defence. The issue was whether the clause was outside the Northern Ireland Assembly’s legislative competence because, without a reasonable excuse defence, it did not enable a fact-specific proportionality assessment and thus could give rise to a disproportionate interference with the article 9, 10 and 11 rights of those wishing to express opposition to abortion services. Giving judgment for the Supreme Court, Lord Reed confirmed an offence can be intrinsically proportionate by way of the ingredients of the offence—and this might be so even without a reasonable excuse defence.^92^ Consequently, a court does not have to go through the process of verifying that a conviction would be proportionate on the facts of every individual case.^93^ In rare instances where the offence’s ingredients do not ensure a conviction’s proportionality, the courts may use section 3 of the HRA to construe the offence so a conviction will always meet the requirements of proportionality (becoming a category 4 offence) or to allow for an assessment of the proportionality of a conviction in the circumstances of individual cases (category 5 offence).^94^

Before moving to explore the rationale for the intrinsically proportionate offences in categories 4 and 6, it is worth observing how the offence-centric paradigm shapes not only the need for a proportionality assessment, but also the threshold question of whether the right is engaged. It does so in two ways. The first is in the context of category 1, where articles 10 and 11 are deemed not to be engaged at all. In *Bauer and Others*, the appellants were convicted of aggravated trespass, contrary to section 68 of the Criminal Justice and Public Order Act 1994, arising from a protest held in Fortnum and Mason over a period of nearly two and a half hours.^95^ Giving judgment for the Divisional Court, Moses LJ observed that the proper starting point in determining whether articles 10 and 11 were engaged was as follows:

It seems to me that it will maintain and protect the rights enshrined in Articles 10 and 11, in the context of peaceful protests, to focus on the question whether those participating in a demonstration are themselves guilty of the conduct element of the crime of aggravated trespass … Since no one suggests that s.68 is itself contrary to either Article 10 or 11, there was no room for any further question or discussion [of breach of Convention rights]. No one can or could suggest that the state was not entitled, for the purpose of preventing disorder or crime, from preventing aggravated trespass as defined in s.68(1).^96^

Similarly, in *Richardson v DPP*, which concerned a conviction for aggravated trespass, the Supreme Court deemed trespass as a limitation on the freedom to protest which is ‘unchallengeably proportionate’.^97^ Lord Hughes simply stated that the offence ‘is not concerned with the rights of the trespasser, whether protester or otherwise’, but rather its concern was ‘a limited class of trespass where the additional sanction of the criminal law has been held by Parliament to be justified … It must be construed in accordance with normal rules relating to statutes creating criminal offences’.^98^ It was just this kind of exclusive focus on the domestic classification of protest activity that led the Dutch Supreme Court astray in *Laurijen and Others* and attracted criticism from Strasbourg.

The second way the scope of articles 10 and 11 has been short-circuited is demonstrated in *AG’s Ref No 1 of 2022*. This concerned four protestors in Bristol charged with criminal damage to a statue of the slave trader Edward Colston.^99^ The Court of Appeal held that section 1(1) of the Criminal Damage Act 1971 encompassed damage outside of the protection of the Convention and, as such, any conviction would be proportionate. However, in what the Court described as a ‘very limited’ number of circumstances, damage construed in the domestic case law as ‘minor’ or ‘temporary’ per section 1(1) could be capable of falling within articles 10(1) and 11(1). In those limited cases, a proportionality assessment of the conviction by the court would be required.^100^ Consequently, whether a conviction for criminal damage requires a proportionality assessment will depend on whether the damaged caused is ‘significant’ (category 2) or ‘minor’/‘temporary’ (category 3). The risk of this binary position is that the proportionality requirement becomes detached from whether the defendant’s conduct is ‘peaceful’ per the ECtHR’s autonomous article 11 meaning because the focus of the criminal court turns instead on whether, as a matter of domestic law, the damage is ‘significant’ or ‘minor’.

### B. The Constitutional Underpinnings

The defining feature of the offence-centric paradigm is that even where articles 10 and 11 are engaged, there is no free-standing duty on a court to be satisfied that a conviction is proportionate on the facts of the individual case. Much will depend on whether, in construing the offence, the courts deem Parliament to have struck the balance between competing rights *a priori*, meaning all convictions, irrespective of the circumstances of the case at hand, will be proportionate. This section argues that beneath the offence-centric paradigm one finds a set of constitutional values concerned with the judiciary’s conception of their role generally, and the criminal court specifically, *vis-à-vis* the legislature. Which institution ought to take the lead role in striking the balance between competing rights or rights that compete with legitimate aims (the institutional question)? And what is the constitutional status of the court as a public authority when it hands down a conviction as a distinct interference with the right to peaceful protest (the status question)?

Taking the ‘institutional question’ first, the offence-centric paradigm involves the judiciary granting deference to Parliament, as the democratically elected institution, to definitively strike the balance between the right to peaceful protest and the rights of others and wider goals of public order. This institutional deference is seen in how the appellate judgments have responded to submissions drawing the courts’ attention to the procedural dimension of articles 10 and 11. Instead of tracing the authorities on protest-related convictions that establish procedural duties on domestic courts, as was done in the previous section, the court have relied on the Grand Chamber’s decision on general measures in *Animal Defenders*.^101^ This case concerned the alleged incompatibility with article 10 of the ECHR of legislation which banned political advertisements on television and radio. In determining whether the substantive right had been breached—so not the procedural duty discussed in section 3—Strasbourg relied on the margin of appreciation to observe that contracting states could, in principle, adopt general measures applicable to predefined situations regardless of individual facts, even if this might result in individual hard cases.

In determining the margin of appreciation in *Animal Defenders*, the ECtHR placed considerable weight on the quality of processes that led to the enactment of the measure:

in order to determine the proportionality of a general measure, the Court must primarily assess the legislative choices underlying it. The quality of the parliamentary and judicial review of the necessity of the measure is of particular importance in this respect, including to the operation of the relevant margin of appreciation.^102^

In *SAZ*, the Supreme Court deemed the general measures in *Animal Defenders* to be analogous to criminal offences and then read the margin of appreciation across to its domestic counterpart, the concept of deference, stating:

Courts therefore have to accord appropriate respect to the assessment made by the decision maker, whether that be Parliament in the case of primary legislation or, in the case of offences created by subordinate or devolved legislation, the government or the devolved legislatures or executives.^103^

Such an account of deference grounded in the democratic legitimacy of the legislature is, of course, a well-established one in a body of constitutional law scholarship too wide-ranging to do justice to here, but worth drawing the relevant connections to.^104^

Put positively, Parliament ought to play a key role in crafting human rights-respecting legislation and making difficult decisions about how to balance competing social goods. Human rights are, after all, as Koskenniemi reminds us, ‘conflicting and contested arguments about the political good’ and ‘dependent on contextual assessments of “proportionality” through which priorities are set among conflicting conceptions of political value and the distribution of scarce resources among contending social groups’.^105^ Parliament is the best institution to take responsibility for generating sufficient consensus as to the kind of harm or levels of disruption caused by civil disobedience and peaceful protest in a democratic society before criminalisation is justified. Writing in the context of criminal law, Kennedy suggests it is the deliberative space and form of public reasoning which the legislative process can provide that gives legitimacy to public decisions to apply criminal censure to certain conduct.^106^ The justification for criminalisation is ‘public’, Kennedy argues, not simply because it is given publicly, but because the form and content of the reasons given enable us to understand, engage and, ultimately, comply with them.

Put negatively, a failure to show deference to Parliament when striking the balance between peaceful protest and public order shifts questions of policy to unelected actors, whether Crown Prosecutors deciding on whether it is in the public interest to prosecute because of protest-related defences, magistrates or Crown Court judges deciding whether, for example, a peaceful environmental protestor’s conviction strikes a fair balance when compared with the general interest of the community. As expressed by Lord Sales writing extra-judicially, ‘the move towards greater fact sensitivity in the form of law can also promote a transfer of control over outcomes from Parliament to other agents. Such a transfer can have clear constitutional implications.’^107^ These implications include the kind of democratic concerns sought to be addressed in clause 7 of the now aborted British Bill of Rights. Resonating to an extent with the offence-centric paradigm’s reference to ‘intrinsic proportionality’, clause 7 sought to require the courts to not only defer, but accept, that Parliament, by having enacted a piece of legislation, decided that the act strikes the appropriate balance between Convention rights or relevant aims, and to give ‘the greatest possible weight’ to the principle that decisions about balancing rights are properly made by Parliament.

The offence-centric paradigm is capable of being further grounded in what has been described by Lord Sales as a rule-of-law argument in favour of ‘bright line rules’ insensitive to individual cases.^108^An age-old tension that confronts legal system is the need, on the one hand, for general rules, spanning a great range of behaviour, that can be applied predictably without requiring guidance or the weighing of socio-political issues and the need, on the other, to leave open for later determination issues which can only be properly appreciated in the context of a specific case.^109^ A common way legislatures resolve this tension is to establish general rules but then leave it to courts (or delegate to administrative bodies) to determine how the open-textured standards or predefined exceptions are to apply in a given case. The adoption of this ‘fact-sensitivity’ within general rules creates space for judges to do justice to an individual’s circumstances which, because of social importance or individual fairness, necessitate a degree of choice in the application of a general rule to a specific case.^110^

The choice of a form of law ‘reflects a deeper choice as to the relative importance in that context of the competing aims of flexibility and certainty’.^111^ Flexibility is offered where a law is capable of greater fact sensitivity in its application, but the cost may be legal certainty and consistency of application. Certainty is provided where a law is determined in abstract and in its application precisely and without resource to individual discretion; the trade-off is that there will inevitably be hard cases and possible injustices. In validating the offences in categories 4 and 6 as existing without the need for a proportionality assessment, the court is prioritising the value of certainty and predictability over the fact-specific flexibility inherent in the justificatory paradigm. As Lord Sales rightly observes, the certainty associated with general rules has the well-recognised advantage of enabling individuals to better plan their affairs in the knowledge of how laws will be applied by decision makers, including the courts.^112^ Strasbourg has accepted that general measures can be a more feasible means of achieving a legitimate aim than a provision allowing a case-by-case assessment, which would risk significant uncertainty, including of litigation, expense and delay, as well as of discrimination and arbitrariness.^113^

Turning more briefly to the ‘status question’, the offence-centric paradigm rests on a further constitutional reading of the criminal court and the nature of a conviction. Section 6(1) of the HRA makes it unlawful for public authorities, including the courts, to act in a way which is incompatible with Convention rights. It is well established that a criminal conviction is a distinct form of interference with articles 10 and 11. *Ziegler* was a reminder of this and its key implication: an interference requires justification of the proportionality of the interference by the relevant public authority.^114^ In *Cuciurean*, the Divisional Court refused to accept section 6 of the HRA as imposing a free-standing obligation on a court to be satisfied that a conviction would be a proportionate interference with Convention rights if that is not an ingredient of a statutory offence. It would, the Court stated, make it impossible for the legislature to enact a general measure which satisfactorily addresses proportionality itself.^115^ In *SAZ*, the Supreme Court confirmed that section 6 does not require a court to be satisfied that a conviction for an offence would be proportionate whenever articles 10 and 11 are engaged. The only avenue for a proportionality requirement as an additional ingredient of a statutory offence was in the case of a category 5 offence, where the offence failed to strike the balance and omitted a reasonable excuse defence, and the requirement for a proportionality assessment stemmed from section 3 of the HRA, not section 6 exclusively.^116^

### C. A Summary of the Offence-centric Paradigm

The offence-centric paradigm is premised on the idea of the conviction as a manifestation of a policy choice by Parliament to criminalise behaviour debated, assessed and justified according to the values and procedures of the political constitution. It is what Möller describes as a conventional process-based justification of a (rights-interfering) law: the justification is it having been passed in line with the democratically elected legislature.^117^ The constitutional implications of this are as follows. First, in construing an offence as intrinsically proportionate, the courts are laying the path for Parliament to prevent scrutiny of the offence’s application to individual cases in circumstances where Parliament is understood by the courts as having struck the balance itself. It is a kind of judicially crafted ouster. Second, the offence-centric paradigm involves accepting a more limited role for courts in protecting human rights within the criminal process.

## 5. In Search of a Compromise

The dilemma is that the offence-centric paradigm reflects a legitimate judicial desire to respect the elected legislature and the legal certainty promoted by fact-insensitive public order offences—but in its current form, categories 4 and 6 put domestic courts at odds with a clear line of Strasbourg authority that necessitates greater judicial scrutiny of protest-related convictions, anchored in a fact-specific proportionality assessment. Working through the dilemma, this final section casts doubt on the coherency of the intrinsically proportionate offences that comprise categories 4 and 6. Having done so, it then proposes how the offence-centric paradigm’s constitutional concerns ought to be properly addressed in a manner that can satisfy the procedural duties of the ECtHR’s justificatory paradigm.

### A. The Incoherencies in Domestic Law

The courts have not yet engaged in a sufficiently close examination of the justificatory paradigm developed in Strasbourg’s article 10 and 11 jurisprudence, nor has there been adequate consideration of its implications for the criminal courts as public authorities per section 6(1) of the HRA. There are three reasons for this lack of engagement, none of which are persuasive.

First, the courts have adopted the standard for reviewing a general measure (eg a rule or policy) from *Animal Defenders* for protest cases where it is not the compatibility of the offence with articles 10 and 11 that is being challenged but its discrete exercise, by way of a conviction on a specific set of facts, as an interference with articles 10 and 11. Because *Animal Defenders* is concerned with the measure generally, ‘integrated procedural review’ makes sense because the object of review is the very provision and its justification on the whole. Accordingly, the quality of the process by which the measure was enacted influences the ECtHR’s level of scrutiny. The adoption of a domestic version of the ‘general measures’ approach to the conviction, thus categorising it as belonging to an intrinsically proportionate offence type, is analytically problematic.

For one reason, it involves conflating these two distinct objects of review: the measure and its application. An offence may be compatible with articles 10 and 11 because it is capable of being applied in most cases in a Convention-compliant manner, yet all the while is applied in practice by prosecuting authorities in a minority of cases in a way which is not. In the words of Lady Arden SCJ in *Ziegler*, when the object of review is not a rule or policy but its application, ‘no proportionality analysis can be conducted in splendid isolation from the facts of the case’.^118^ Category 4 convictions deny this reality by preventing an evaluation of proportionality where the offence’s application may turn out to be disproportionate on a specific set of facts. For another reason, a regulatory measure governing political advertising in the broadcasting industry is not analogous to criminal offence that risks stigma and sanction in the first place. In *Perinçek*, which developed article 10’s procedural limb, the Grand Chamber expressly described *Animal Defenders* as concerning a ‘regulatory scheme’ to be viewed ‘by contrast’ to a criminal conviction—‘one of the most serious forms of interference with the right to freedom of expression’.^119^ Most recently, Strasbourg observed:

Where the sanctions imposed on the demonstrators are criminal in nature, they require particular justification. A peaceful demonstration should not, in principle, be rendered subject to the threat of a criminal sanction, and notably to deprivation of liberty. Thus, the Court must examine with particular scrutiny the cases where sanctions imposed by the national authorities for non-violent conduct involve a prison sentence.^120^

The appellate courts’ reliance on *Animal Defenders* is further problematic because it does not translate to the domestic context. This style of procedural review involves the ECtHR reviewing the quality of legislative discussion and scrutiny of general measures to determine how much weight to give the balance struck by national authorities. The domestic version of this would be courts reviewing the quality of parliamentary debate, committee reports, White Papers etc. Yet this would strike at the heart of article 9 of the Bill of Rights 1688, which prevents courts from determining the adequacy or cogency of any parliamentary consideration of Convention rights.^121^ Accordingly, domestic courts have adopted a much scaled-back, deferential, domestic version of Strasbourg’s integrated review in domestic law for legislation interfering with qualified rights where Parliament might have struck the balance itself. When reviewing the proportionality of a general measure, including criminal offences, the courts will consider it relevant if Parliament did form a judgment that the legislation was appropriate, notwithstanding its potential impact on Convention rights; however, the absence of such consideration will not count against upholding the compatibility of the measure.^122^

The result is a lacuna in accountability for rights interferences in the criminal process. The risk is that neither the criminal offence nor its application on the facts of the case is subject to a rigorous proportionality assessment by the courts. In a keenness to contain *Ziegler*, the strong approval of ‘intrinsically proportionate’ offences in *Cuciurean* and *SAZ* gives rise to a kind of *de facto* ouster clause—one brought into being by the judiciary. The proportionality of the defendant’s conviction will never be justified on the individual facts of the case because the offence itself is deemed by the courts to have struck a proper balance between competing rights. And by adopting an *Animal Defenders* style of review rather than the procedural limbs of articles 10 and 11, the courts are unable to meaningfully reviewing whether, and if so how, such a balance was struck by Parliament in the first place. Is this dual immunity from scrutiny of an offence *and its application* where articles 10 and 11 are engaged really something Parliament intended when it legislated for offences in category 4? If it was, Parliament would have been expected to do so expressly.

Second, the domestic courts have yet to consider the full bodies of Strasbourg case law that establish the procedural duties as part of articles 10 and 11, including the cases handed down by Strasbourg in 2022 and 2023. In *AG’s Ref No 1 of 2022*, the Court of Appeal dealt with *Handzhiyski* and *Perinczek*, mentioned above, in isolation from the rest of the article 10 and 11 procedural case law, relying on the narrowest of grounds to dismiss their applicability. The reference to the procedural limb in *Handzhiyski* was dismissed by the Court of Appeal because the obligation on domestic courts was caveated with the words ‘normally’ and ‘so cannot be said to lay down a clear-cut rule’—overlooking the subsequent sentence that an interference in the form of a penalty ‘inevitably calls for a detailed assessment of the specific conduct sought to be punish’.^123^ As for *Perinczek*, the Court of Appeal baldly stated without explanation that the ‘stricter scrutiny’ Strasbourg required for criminal convictions where the right to peaceful expression was engaged did not necessarily involve a fact-specific proportionality assessment, but without stating what form ‘stricter scrutiny’ should take instead. The Supreme Court in *SAZ* similarly observed that:

its [the European Court of Human Rights] task is not to review legal provisions and practice in abstracto, but to determine whether the manner in which they were applied to or affected the applicant gave rise to a violation of the Convention. Domestic courts are not required to proceed on the same basis.

The issue is that the procedural limbs in articles 10 and 11 *do* require domestic courts to proceed on a similar basis to Strasbourg by satisfying the twin procedural requirements outlined earlier.

Third, regarding the duties of a criminal court under section 6(1) of the HRA where articles 10 and 11 are engaged, there has been relatively limited judicial consideration of this section’s scope and significance. The HRA is a ‘constitutional instrument’.^124^ It seems strange, as a matter of constitutional ordering, to suggest, as the High Court does in *Cuciurean*, that the court’s duty under section 6 of the HRA is dependent, first, on substantive criminal offence arising from ‘ordinary’ legislation and, second, on the basis of offences enacted years, if not decades, prior to the HRA. No doubt when *legislation* is deemed to raise issues of compatibility, it is section 6 that requires the court as a matter of law to turn to section 3 or 4 as a constitutional tool, but if one accepts that the application of the legislation—the *conviction*—is an interference with articles 10 and 11 that is distinct from the decision to prosecute, as the ECtHR did in *Steel* and the Supreme Court did in *Ziegler*, then the same is not true in reverse: just because the court does not deem it necessary to use section 3 or 4, that does not extinguish section 6. That is to say, section 6 has been, but should not be, relegated to a conduit for sections 3 and 4; it is surely a provision that prohibits a criminal court from exercising its power to convict in a manner that would be disproportionate on the facts of the case before it.

Another way of looking at the section 6 duty on courts is to take seriously Lord Kerr’s observation in *Finucane* that a failure to acknowledge a breach of a Convention right would ‘be in breach of the spirit, if not the literal requirement, of section 6(1) of the HRA. This is particularly so because of section 6(6) of HRA. It stipulates that an act includes a failure to act.’^125^ There would be, on this article’s analysis, a breach of the Convention where the court fails to satisfy the procedural duties under articles 10 and 11 to conduct a fact-specific proportionality assessment. Indeed, when looked at holistically, the sidelining of section 6’s application to the criminal court handing down the conviction creates an odd lacuna in the HRA’s influence on the criminal process. Police must exercise powers of arrest, detention and restrictions relating to peaceful protest in a way that is proportionate to the right to peaceful protest.^126^ The Crown Prosecution Service (CPS) must exercise prosecutorial discretion in a Convention-compliant manner.^127^ The sentencing judge must punish the offender in a manner proportionate to articles 10 and 11.^128^ Yet there is no free-standing obligation on a criminal court to justify its own interference with the right—the conviction—as proportionate on the facts before it.

### B. A Proposed Compromise

A critical examination of domestic law will now most likely depend on a successful challenge to a conviction arising from an offence in category 4 or 6 at Strasbourg, or a reconsideration of *SAZ* by the Supreme Court in light of the ECtHR’s jurisprudence discussed in section 3. In considering the implications of Strasbourg’s justificatory paradigm, though, much will hinge on the duty of domestic courts under section 2(1) of the HRA to ‘take into account’ the ECtHR jurisprudence. The classic formulation of the duty—the ‘mirror principle’—is that domestic courts should follow any clear and constant line of ECtHR jurisprudence^129^ and keep pace with its evolution ‘no more, but certainly no less’.^130^ As Lord Reed observed in *R (Elan-Cane)*, the articles of the Convention have the same content at the domestic level as at the international one, so ‘should in principle’ receive the same interpretation at both levels.^131^ But in reality the courts have considerable discretion to depart from Strasbourg should they wish to do so.^132^ Termed the ‘partial mirror’ approach by Fenwick and Masterman,^133^ there has been a divergence from the ECtHR’s case law where domestic courts consider there are ‘good’^134^ or ‘strong‘^135^ reasons for doing so.

The risk is that the courts evade the critique just outlined and maintain the misguided *Animal Defenders* approach, seeking refuge in an expansive reading of section 2(1) of the HRA. A possible charge is that the article 10 and 11 procedural duties ought not to be followed because—drawing on the ‘semi-mirror’ jurisprudence—Strasbourg has ‘insufficiently appreciated aspects of our domestic process’^136^ or ‘overlooked or misunderstood some argument or point of principle’^137^ that is fundamentally at odds with the ‘distribution of powers’.^138^ Here, one might see the constitutional values that underpin the offence-based paradigm come to the fore. The procedural duties of articles 10 and 11 are further vulnerable to blunt refusals to follow Strasbourg where domestic courts are dissatisfied with the ECtHR’s reasoning, in disagreement with the conclusion or concerned with the decision’s practical implications.^139^ However, for a departure from Strasbourg’s justificatory paradigm to be convincing, it must somehow answer the three critiques just outlined—not least the far greater complexity of translating the *Animal Defenders* approach, upon which the offence-based paradigm now seems to rest, to the domestic constitutional context.

Just as fundamentally, though, proponents of a divergence from Strasbourg must explain why the course correction proposed in the proceeding paragraphs—which was adopted by the Supreme Court having considered section 2(1) in a similar constitutional context—cannot remedy concerns of principle as well as practical operation. What I want to demonstrate now is that there is a way of domesticating the procedural duties Strasbourg requires, using statutory mechanics already on offer in the HRA. This compromise is, it is argued, sensitive to the constitutional issues of deference, as well as practical concerns about its operation at trial. This would require domestic courts to embark on two steps: first, to detach the dependence of proportionality assessments in individual cases from the ingredients of the offence and attach it instead to a legal basis that will enable such assessments as a matter of principle; and second, to adapt the test of proportionality so as to operate in a manner responsive to the constitutional context in which it is being applied (discussed in the following subsection).

Section 6 of the HRA should be the legal basis upon which to ground a standalone public law duty requiring the first instance court to ensure that its exercise of power, in the form of a conviction, is a justified interference with the rights of the defendant on the facts. Just as a first instance court can be subject to judicial review on public law grounds—the court cannot, for example, arrive at an outcome that is *Wednesbury* unreasonable—section 6 should also be read as making it unlawful for a criminal court to arrive at a decision which would amount to a disproportionate interference with articles 10 and 11. This is not a remarkable proposition. It will be recalled that section 6(3)(a) expressly includes courts as public authorities for the purposes of the HRA. As Lord Nicholls observed in *Kay v Lambeth City Council* on the scope of section 6: ‘Courts are bound to conduct their affairs in a way which is compatible with Convention rights. The court’s own practice and procedures must be Convention-compliant.’^140^ In the words of Lord Kerr, ‘Reticence by the courts of the UK to decide whether a Convention right has been violated would be an abnegation of our statutory obligation under section 6 of HRA’.^141^ The offence-centric paradigm’s refusal to leave it open to a defendant, as a matter of principle, to raise an article 10 or 11 proportionality-based defence on the facts of their particular case means the practice in category 4 and 5 offences is one in which the court will never itself have justified its interference with the right.

An alternative legal basis for grounding the free-standing proportionality assessment is section 7(1) of the HRA. Section 7(1) allows a person to rely on Convention rights in any legal proceedings where that person claims that a public authority has acted (or proposes to act) in a way which is made unlawful by section 6(1). Legal proceedings expressly include those brought by or at the instigation of a public authority.^142^ This provision has been entirely overlooked, but it is not clear why. A criminal trial can be understood as falling within section 7(1). By determining whether to convict on the ingredients of the offence alone, in the absence of consideration of the defendant’s article 10 or 11 rights, the court would be ‘proposing’ to act in a way contrary to section 6(1). The court, as a public authority, would have failed to satisfy the procedural safeguards of articles 10 and 11, which is sufficient grounds to breach the rights and thus to have acted in a way incompatible with the Convention. Alternatively, the public authority requirement is satisfied by the role of the CPS at trial which, in prosecuting the defendant and seeking a conviction for conduct protected by articles 10 and/or 11, is acting unlawfully. As for section 7(2), a criminal trial is a proceeding brought by a public authority—the CPS. The necessary implication of the broad definition of ‘legal proceedings’ is that criminal proceedings fall within section 7(1).

### C. A Modified Proportionality Test

Even if either of these statutory bases can ground a free-standing duty on domestic courts to conduct a fact-specific proportionality assessment, how is this to be reconciled with the values of legal certainty and democratic legitimacy that underpin the offence-based paradigm? The answer is to be found in the adaption of proportionality crafted once before by the Supreme Court. This was done in the context of claims for possession by local authorities that engaged the article 8 ECHR rights of the residential occupier. The statutory scheme that established local authorities’ powers to seek possession of public housing was carefully designed by Parliament.^143^ It was grounded in sound reasons of social policy and purposefully empowered authorities to administer limited housing stock in the interests of the community.^144^ In three cases, the House of Lords held that the statutory scheme did not permit the occupier to raise a proportionality defence at possession proceedings.^145^ However, in *Kay v UK*, the ECtHR found this outcome to be in violation of article 8’s procedural safeguard, which requires that a person at risk of losing their home should, in principle, be able to have the proportionality of the measure determined by an independent tribunal in light of the relevant principles.^146^

In *Manchester CC v Pinnock*, the Supreme Court sought to give effect to *Kay* and the article 8’s procedural duty by adapting domestic law accordingly: where an order for possession of a person’s home is at the suit of a local authority, the county court must have the power to assess the proportionality of making the order.^147^ The Supreme Court considered it would be wrong not to follow the ECtHR’s jurisprudence per section 2(1) of the HRA.^148^ In requiring that a county court have the ability to engage in a fact-specific proportionality assessment, there was no question of Strasbourg cutting across domestic substantive or procedural law in some fundamental way.^149^ Instructive for our analysis here is not only the willingness to follow Strasbourg jurisprudence, but also how, in *Manchester CC* and the sequel, *Hounslow LBC v Powell*,^150^ the Supreme Court harnessed the malleability of proportionality itself. A free-standing proportionality assessment was devised in a way that remained sensitive to Parliament in this area of social policy, as well as the need to prevent county court possession proceedings being derailed by unmerited article 8 violation claims.

The Supreme Court achieved this with three focused adaptions in the context of the county court to the application to the *Bank Mellat* proportionality test.^151^ First, the issue of proportionality must be raised by the occupier.^152^ Second, if it was raised, the court should initially consider it summarily and, if satisfied that, even if the facts are made out, the point would fail, it should be dismissed.^153^ Only if the court was satisfied that the proportionality point is seriously arguable enough that it could affect the court’s decision should the point be further entertained. If the court decided to entertain the proportionality point because it is seriously arguable, it must give a reasoned decision as to whether a fair balance would be struck by the order for possession. Third, there should be an assumption that the legitimate aims of the local authorities in seeking possession are satisfied.^154^ The aims will almost always be to give effect to the social policies in the statutory scheme which are legitimate ones for the purposes of the Convention. The focus of the county court, therefore, would be on the occupier’s personal circumstances and any factual objections they raise, with the question of whether the order would be proportionate in the individual case.^155^

There is a strong argument for domesticating the procedural duties under articles 10 and 11 for prosecutions of peaceful protestors in a similar fashion to article 8 in the housing context. As a matter of principle, there are similar types of constitutional considerations at play for evictions and convictions. For both, Parliament has sought through legislation to give effect to public policy concerns—in the former, social policy; in the latter, public order and safety—and these will almost invariably satisfy a legitimate aim of articles 10(2) and 11(2). Likewise, statutory schemes for evictions and convictions require non-judicial actors to make preliminary yet pivotal decisions about the balance to be struck in individual cases: local authorities in the case of evictions, the CPS in the case of convictions. As a matter of practicality, concerns about trials being derailed and distracted by proportionality assessments in the magistrates’ court have been raised in the wake of *Ziegler*.^156^ The modified proportionality assessment in *Manchester CC* and *Hounslow LBC* was designed to guard against prolonged, expensive litigation by preventing at an early-stage spurious proportionality arguments being made and diverting limited resources of public authorities.^157^ By placing the onus on the defendant to raise the disproportionality issue, by requiring the proportionality point to be seriously arguable and by assuming the conviction pursues a legitimate aim, issues of principle and practicality can be addressed in the criminal context, as they have been in the civil, while honouring the procedural dimensions that ensure the protection of articles 10 and 11.

Practically, if this middle ground were adopted, prosecutions arising from offences in categories 4 and 6 would require that the criminal court be satisfied, on the basis of the modified fact-sensitive assessment just discussed, that the conviction is proportionate. This would concern defendants prosecuted for breaching conditions imposed under sections 12 and 14 of the Public Order Act 1986, violating the restrictions placed on protests within the vicinity of abortion centres,^158^ and, if the Home Office succeeds in its amendments to the Criminal Justice Bill, those charged with the newer offences of ‘locking on’ and ‘obstructing public highways’ in the Public Order Act 2023.^159^ A sceptic might ask what difference this middle course would really make. If the criminal courts interpret the ‘seriously arguable’ standard too strictly, the procedural duties on the court risk being devoid of any real meaning. Similarly, the presumption of a legitimate aim could risk clouding the court’s proportionality assessment, tipping the scales against the rights holder from the outset. Further still, placing the presumption to raise the point on the defendant sits uncomfortably with the burden of proof resting on the prosecution and the state’s duty to justify interferences with the right. Should the approach proposed here ever materialise, it is well worth being vigilant of such risks.

## 6. Conclusion

Must a criminal court conduct a fact-sensitive proportionality assessment where a defendant’s conviction for a public order offence would amount to an interference with their right to peaceful protest? This article has exposed how and why the ECtHR and domestic courts are arriving at different answers to this fundamental question. This involved tracing the doctrinal developments of both sets of courts, but also setting these developments within the deeper constitutional moulds to which they belong. Two paradigms emerged from this analysis: the ‘justificatory paradigm’ authored by Strasbourg and the ‘offence-centric paradigm’ crafted by domestic courts. This conceptualisation, it has been argued, allows one to make sense of, and begin to disentangle, the legal knots of criminal law, human rights law and public law that are closely interwoven in this area.

Doctrinally, the article has revealed that the approach to convicting peaceful protestors in England and Wales is diverging from what the ECtHR now expects of national courts. Domestic law has yet to acknowledge the presence and implications of the procedural duties under articles 10 and 11. These *do* require domestic courts to justify convictions on the facts of individual cases as a matter of principle. This poses a challenge to the domestic appellate courts’ decision to interpret offences—those in categories 4 and 6—so as to foreclose the criminal court’s ability to assess the proportionality of a conviction on the circumstances of the specific case before it.

Conceptually, the constitutional values that underpin each paradigm were brought to the surface. Doing this enabled a better grasp of each paradigm’s relative concern with the role and significance of the proportionality test where articles 10 and 11 are engaged. It revealed the fault lines that mark the diverging approaches of Strasbourg and domestic courts to be principled ones, animated by concerns over: the proper justificatory function of courts in conducting rights-based adjudication; the extent of deference that ought to be shown by courts to the legislature in crafting public order offences; and the nature of a criminal conviction as a judicially imposed interference with protest rights.

It is by discussing these constitutional values alongside the case law that one can start to imagine a way of satisfactorily addressing the divergence between the two paradigms. It was argued that where articles 10 and/or 11 are engaged by a conviction, the Supreme Court should embrace a standalone proportionality assessment, albeit one carefully calibrated in a manner similar to that in *Manchester CC* and *Hounslow LBC*. This would respect Parliament’s proper role in crafting public order offences, while guarding against the risk of applying offences in rights-infringing situations never fully envisioned, let alone considered, by Parliament.

## Footnotes

1 In the domestic law: *Director of Public Prosecutions v Cuciurean* [2022] EWHC 736 (Admin); *Attorney General’s Reference No 1 of 2022* [2022] EWCA Crim 1259; *Attorney General for Northern Ireland—Abortion Services (Safe Access Zones) (Northern Ireland) Bill* [2022] UKSC 32 (*SAZ*); *DPP v Eastburn* [2023] EWHC 1063 (Admin)*; Hicks v DPP* [2023] EWHC 1089 (Admin). In the European human rights law: *Öğrü and others v Turkey* App no 60087/10 (28 May 2018); *Obote v Russia* App no 58954/0 (19 February 2020); *Laurijsen and Others v the Netherlands* App no 56896/17 (21 November 2023); *Bumbeș v Romania* App no 18079/15 (3 May 2022)

2
*SAZ* (n 1) [35]–[41], [64].

3 The trend of criminalising peaceful protest, especially in context of environmental activists, extends beyond England and Wales to democracies across Europe. See Jan-Werner Müller, ‘Protest Problems’ *London Review of Books* (8 February 2024); Michael Forst, ‘State Repression of Environmental Protest and Civil Disobedience: A Major Threat to Human Rights and Democracy’ (Position Paper, UN Special Rapporteur on Environmental Defenders under the Aarhus Convention, February 2024).

4 eg *Cuciurean* (n 1).

5 eg *AG’s Ref No 1 of 2022* (n 1).

6 Public Order Act 1986 (Serious Disruption to the Life of the Community) Regulations 2023, s 2(1); and see ‘Police to Be Given Clearer Powers on Slow-Walk Protests’ BBC News (13 June 2023) <www.bbc.co.uk/news/uk-politics-65897513>.

7 Public Order Act 1986, s 12(aa).

8 Public Order Act 2023, ss 1, 6–7.

9 For discussion of these components, see Aharon Barak, *Proportionality: Constitutional Rights and their Limitations* (CUP 2012); Kai Möller, *The Global Model of Constitutional Rights* (OUP 2012) ch 7.

10 Phillip Sales and Ben Hooper, ‘Proportionality and the Form of Law’ (2003) 119 LQR 426.

11 See *City of London Corpn v Samede* [2012] EWCA Civ 16 (Lord Neuberger); *Director of Public Prosecutions v Ziegler and others* [2021] UKSC 23 [74]–[78] (Lords Hamblen and Stephens).

12 See Singh LJ in *DPP v Ziegler and others* [2019] EWHC 71 [84]–[86].

13 The jurisprudence is set at out length in s 3.

14 Mattias Kumm, ‘The Idea of Socratic Contestation and the Right to Justification: The Point of Rights-Based Proportionality Review’ (2010) *4* Law & Ethics of Human Rights 140; Moshe Cohen-Eliya and Iddo Porat, ‘Proportionality and the Culture of Justification’ (2011) Am J Comp L 59(2) 463, 479.

15 The case law is set out in detail in s 4.

16
*SAZ* (n 1) [55]–[56].

17
*Animal Defenders International v UK* App no 48876/08 (22 April 2013).

18 Kai Möller, ‘Justifying the Culture of Justification’ (2019) 17(4) ICON 1078, 1093; Cohen-Eliya and Porat (n 14).

19 Kumm (n 14).

20 Möller, ‘Justifying the Culture’ (n 18).

21 Kumm (n 14) 144.

22 ibid.

23 Helen Fenwick, ‘The Right to Protest, the Human Rights Act and the Margin of Appreciation’ (1999) MLR 62; Joint Committee on Human Rights, *Legislative Scrutiny: Public Order Bill* (2022–23, HL 16, HC 351); *Cuadrilla Bowland Ltd & Ors v Lawrie & Ors* [2020] EWCA Civ 9.

24
*Oberschlick v Austria (no 1)* (1991) 19 EHRR 389, § 57; *Woman on Waves and Others v Portugal* App no 31276/05 (ECtHR, 3 February 2009) § 30; *Bumbeș* (n 1) 63.

25
*Rai and Evans v UK* App no 26258/07 (17 November 2009); *Frumkin v Russia* App no 74568/12 (5 January 2016); *Annenkov and Others v Russia* App no 31475/10 (25 October 2017); *Öğrü* (n 1); *Peradze and others v Georgia* App no 5631/16 (15 March 2023).

26 Cohen-Eliya and Porat (n 14) 483.

27
*Sergey Kuznetsov v Russia* App no 10877/04 (23 January 2009) § 45; *Alekseyev v Russia* App no 4916/07 (11 April 2011) § 80; *Tatár and Fáber v Hungary* App no 26005/08 (12 June 2012) § 37; *Gün and Others v Turkey* App no 8029/07 (18 June 2013) § 49; *Taranenko v Russia* App no 19554/05 (13 October 2014) § 66.

28
*Steel and Others v UK* App no 24838/94 (23 September 1998) § 92.

29
*Barraco v France* App no 31684/05 (5 June 2009).

30
*Ekrem Can and Others v Turkey* App no 10613/10 (5 September 2022).

31
*Kudrevičius and Others v Lithuania* App no 37553/05 (15 October 2015).

32
*Laurijsen* (n 1) §§ 54–5.

33
*Tatár* (n 27) § 38.

34 ibid § 38.

35
*Primov and Others v Russia* App no 17391/06 (12 June 2014) § 119; *Novikova and Others* App no 25501/07 (12 September 2016) §163; *Kudrevičius* (n 31) § 155; *Obote* (n 1) § 42.

36
*Laurijsen* (n 1) §§ 65–6.

37 Cohen-Eliya and Portat (n 14) 480.

38
*Handyside v UK* App no 5493/72 (7 December 1976) § 65.

39
*Sunday Times v UK* (A/30) (1979–80) (26 April 1979) § 65.

40 ibid.

41
*Oberschlick* (n 24) § 60, emphasis added.

42
*Schwabe v Austria* App no 13704/88 (8 January 1991); *Jersild v Denmark* App no 15890/89 (23 September 1994); *Christine Goodwin v UK* App no 17488/90 (27 March 1996); *Zana v Turkey* App no 18954/91 (25 November 1997)

43
*Perinçek v Switzerland* App no 27510/08 (15 October 2015).

44 ibid § 278.

45 ibid § 273, emphasis added.

46
*Saygılı and Karataş v Turkey* App no 51041/99 (16 April 2018) § 30.

47
*Handzhiyski v Bulgaria* App no 10783/14 (6 July 2021).

48 ibid § 52, emphasis added.

49 ibid § 55.

50
*Bumbe*ș (n 1).

51 ibid § 98.

52
*United Communist Party of Turkey and Others v Turkey* [1998] Reports of Judgments and Decisions 1998-I, § 47.

53
*Primov* (n 35) § 145; *Kudrevičius* (n 31); *Novikova* (n 35) § 165; *Annenkov* (n 25); *Bumbe*ș (n 1) § 95.

54
*Öğrü* (n 1); *Obote* (n 1); *Bumbe*ș (n 1); *Laurijsen* (n 1).

55
*Öğrü* (n 1).

56 ibid § 67. The judgment is in French; the extract is based on a translation.

57
*Zehentner v Austria* App no 20082/02 (16 October 2009).

58
*Bjedov v Croatia* App no 42150/09 (29 August 2012).

59 See eg Kumm (n 14); Cohen-Eliya and Porat (n 14); Möller, ‘Justifying the Culture’ (n 18).

60 Kumm (n 14) 169.

61 ibid.

62 ibid 168.

63 Earliest references are *Handyside* (n 38) § 49; *Barthold v Germany* App no 8734/79 (25 March 1986) § 58; *G v Germany* App no 13079/87 (14 March 1989); *Bédat v Switzerland* App no 56925/08 (29 March 2016) § 48. As for art 11, see *Rai, Allmond and ‘Negotiate Now’ v UK* App no 25522/94 (6 April 1995).

64
*Eva Molnár v Hungary* App no 10346/05 (1 July 2009) § 42.

65 Office for Democratic Institutions and Human Rights and Venice Commission, ‘Guidelines on Freedom of Peaceful Assembly’ (3rd edn, July 2019) CDL-AD(2019)017.

66 Cohen-Eliya and Porat (n 14) 483.

67 ibid.

68 Kumm (n 14) 160–1.

69 ibid.

70 Cited in Joint Committee on Human Rights, *Legislative Scrutiny: Bill of Rights Bill* (2022–23, HL 132, HC 611) para 142.

71 David Ormerod, ‘In Protest against Ziegler’ (2022) Crim LR 427; Richard Martin, ‘The Public Order Provisions of the Police, Crime, Sentencing and Court Bill: A “Modest Reset of the Scales”?’ (2021) Crim LR 12 1008.

72 Kumm (n 14) 161.

73 Martin (n 71).

74 Michael Forst, ‘Visit to London, United Kingdom of Great Britain and Northern Ireland, 10–12 January 2024’ (end of mission statement, UN Special Rapporteur on Environmental Defenders under the Aarhus Convention, 23 January 2024).

75
*Norwood v DPP* [2002] EWHC 1564 (Admin); *Hammond v DPP* [2004] EWHC 69; *Abdul v DPP* [2011] EWHC 247 (Admin).

76
*Bauer v DPP* [2013] EWHC 634 (Admin); *James v DPP* [2015] EWHC 3296 (Admin).

77
*Cuciurean* (n 1); *AG’s Ref No 1 of 2022* (n 1); *Eastburn* (n 1).

78 Davis LJ in *James* (n 76) summarising the case law at [51].

79
*Norwood* (n 75) [35]; *Hammond* (n 75) [23]; *Abdul* (n 75) [56].

80
*James* (n 76).

81
*Eastburn* (n 1).

82
*James* (n 76) [35].

83 ibid [39]–[42].

84 Home Office, ‘New Protest Laws on Face Coverings and Pyrotechnics’ (8 February 2024).

85
*Ziegler* (n 11).

86 ibid [92].

87 ibid [70].

88 ibid [57].

89 ibid.

90
*Cuciurean* (n 1) [67].

91 ibid [69].

92 ibid [52], [62], [78]; *AG’s Ref No1 of 2022* (n 1) [78].

93
*Cuciurean* (n 1) [55]; *AG’s Ref No1 of 2022* (n 1) [42].

94
*SAZ* (n 1) [58].

95
*Bauer* (n 76).

96 ibid [38]–[39].

97
*Richardson and another v Director of Public Prosecutions* [2014] UKSC [83].

98 ibid.

99
*AG’s Ref No 1 of 2022* (n 1).

100 ibid [116].

101
*Cuciurean* (n 1)*; AG’s Ref No 1 of 2022* (n 1); *SAZ* (n 1).

102
*Animal Defenders* (n 17) § 108.

103
*SAZ* (n 1) [55].

104 eg TRS Allan, ‘Human Rights and Judicial Review: A Critique of “Due Deference”’ (2006) 65(3) CLJ 671; AL Young, ‘In Defence of Due Deference’ (2009) 72(4) MLR 554.

105 Martti Koskenniemi, ‘The Effect of Rights on Political Culture’ in P Alston (ed), *The EU and Human Rights* (OUP 1999), cited in Conor Gearty, *Principles of Human Rights Adjudication* (OUP 2005) 29.

106 Jeffrey Kennedy, ‘Crimes as Public Wrongs’ (2021) 27 Legal Theory 253.

107 Lord Sales, ‘The Developing Jurisprudence of the Supreme Court on Convention Rights’ (Keynote Speech, Scottish Public Law Group Conference, 5 June 2023) para 34.

108 ibid.

109 HLA Hart, *The Concept of Law* (2nd edn, Clarendon Press 1994) 130; William Lucy, ‘Abstraction and the Rule of Law’ (2009) 29(3) OJLS 481.

110 Lucy (n 109).

111 Sales (n 107) para 37.

112 ibid.

113
*Animal Defenders* (n 17) § 108.

114
*Ziegler* (n 11) [70].

115
*Cuciurean* (n 1) [71].

116
*SAZ* (n 1) [56]–[57].

117 Möller, ‘Justifying the Culture’ (n 18) 1088.

118
*Ziegler* (n 11) [108].

119
*Perinçek* (n 43) §§ 272–3.

120
*Peradze* (n 25) § 35; *Ekrem* (n 30) § 92.

121
*R (SC) v Secretary of State for Work and Pensions* [2022] UKSC 26 [164]–[165].

122 ibid [182]; *AG’s Ref No 1 of 2022* (n 1) [62].

123 The same reasoning was used to swiftly dismiss *Handzhiyski* in *SAZ*.

124
*R (HS2 Action Alliance Limited) v Secretary of State for Transport and another* [2014] UKSC 3 [207].

125
*Re Finucane* [2019] UKSC 7, [152].

126
*R* (*Laporte) (FC) v Chief Constable of Gloucestershire* [2006] UKHL 55.

127
*R v Waya* [2012] UKSC 51 [19].

128
*Roberts v R* [2018] EWCA Crim 2739.

129
*R (Alconbury Developments Ltd) v Secretary of State for the Environment, Transport and the Regions* [2001] UKHL 23 [26].

130 R *(Ullah) v Special Adjudicator* [2004] UKHL 26, [20].

131
*R (Elan-Cane) v Secretary of State for the Home Department* [2021] UKSC 56, [87].

132 Lewis Graham, ‘Taking Strasbourg Jurisprudence into Account’ (2022) 2 EHRLR 163.

133 Helen Fenwick and Roger Masterman, ‘The Conservative Project to “Break the Link between British Courts and Strasbourg”: Rhetoric or Reality?’ (2017) 80(6) MLR 1111.

134
*R (Elan-Cane)* (n 131) [101].

135
*R (Ullah)* (n 130) [20].

136
*R v Horncastle* [2009] UKSC 14 [11].

137
*Manchester City Council v Pinnock* [2011] UKSC 6 [48].

138
*Alconbury* (n 129) [76].

139 For detailed discussion of these cases, see Graham (n 132); Fenwick and Masterman (n 133).

140
*Kay v Lambeth City Council; Leeds City Council v Price* [2006] UKHL 10 [61].

141
*Commissioner of Police of the Metropolis v DSD and Anor* [2018] UKSC 11 [78].

142 HRA, s 7(6)(b).

143 Housing Act 1996, Part VII.

144
*Hounslow LBC v Powell; Leeds CC v Hall; Birmingham CC v Frisby* [2011] UKSC 8 [41].

145
*Harrow London Borough Council v Qazi* [2003] UKHL 43; *Kay v Lambeth London Borough Council* [2006] UKHL 10; *Doherty v Birmingham City Council* [2008] UKHL 57.

146
*Kay v UK* App no 37341/06 (21 December 2010).

147
*Manchester CC* (n 137).

148 ibid [48].

149 ibid [49].

150
*Hounslow LBC* (n 144).

151
*Bank Mellat v Her Majesty’s Treasury (No 1)* [2013] UKSC 38.

152
*Manchester CC* (n 137) [61].

153
*Hounslow LBC* (n 144) [33].

154
*Manchester CC* (n 137) [53].

155
*Hounslow LBC* (n 144) [37].

156 Ormerod (n 71).

157
*Hounslow LBC* (n 144) [41].

158 Abortion Services (Safe Access Zones) Act (NI) 2023, s 5(1); POA 2023, s 9(1).

159 Richard Martin, ‘The Public Order Act 2023’ *Blackstone’s Briefing* (forthcoming).

